# Tris(dibenzoyl­methanido-κ^2^ *O*,*O*′)[(6*S*,8*S*)-(+)-7,7-dimethyl-3-(2-pyrid­yl)-5,6,7,8-tetra­hydro-6,8-methano­isoquinoline-κ^2^ *N*,*N*′]gadolinium(III)

**DOI:** 10.1107/S1600536809030700

**Published:** 2009-08-08

**Authors:** Xi-Li Li, Lai-Fu He

**Affiliations:** a Henan Provincial Key Laboratory of Surface & Interface Science, School of Materials and Chemical Engineering, Zhengzhou University of Light Industry, Henan, Zhengzhou 450002, People’s Republic of China

## Abstract

In the title compound, [Gd(C_15_H_11_O_2_)_3_(C_17_H_18_N_2_)], the Gd^III^ atom is coordinated by six O atoms from three β-diketonate ligands and two N atoms from a chiral ligand L_*S*_,_*S*_-(+)-7,7-dimethyl-3-(2-pyrid­yl)-5,6,7,8-tetra­hydro-6,8-methano­iso­quinoline, in a coordination geometry best described as distorted square-anti­prismatic.

## Related literature

For general background, see: Kaneko *et al.* (2006 ▶); Kimura *et al.* (2003 ▶). For related structures, see: Li *et al.* (2007 ▶). For the synthesis, see: Carles & Ohlmann (1965 ▶); Hayoz & Zelewsky (1992 ▶).
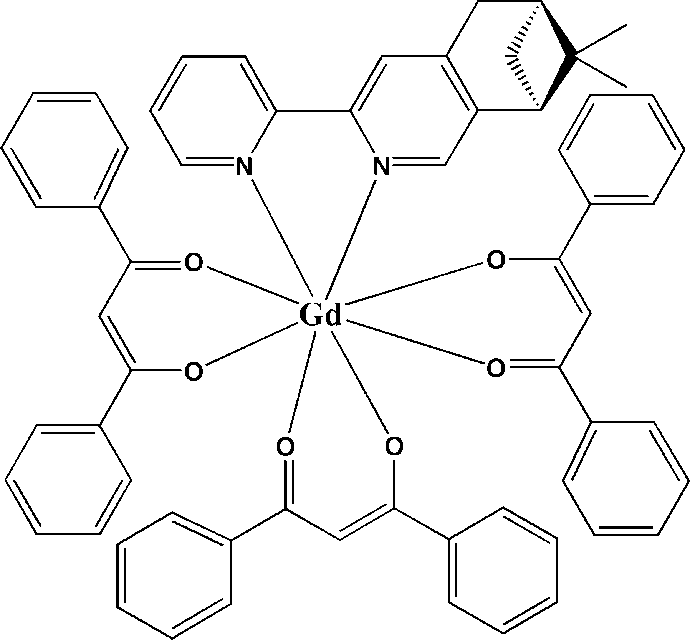


## Experimental

### 

#### Crystal data

[Gd(C_15_H_11_O_2_)_3_(C_17_H_18_N_2_)]
*M* *_r_* = 1077.30
Monoclinic, 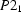

*a* = 9.5303 (19) Å
*b* = 20.814 (4) Å
*c* = 12.735 (2) Å
β = 92.421 (4)°
*V* = 2523.9 (8) Å^3^
*Z* = 2
Mo *K*α radiation
μ = 1.37 mm^−1^
*T* = 298 K
0.30 × 0.24 × 0.22 mm

#### Data collection

Bruker SMART APEX CCD diffractometer
Absorption correction: multi-scan (*SADABS*; Bruker, 2001 ▶) *T*_min_ = 0.680, *T*_max_ = 0.742
13247 measured reflections
9009 independent reflections
8750 reflections with *I* > 2σ(*I*)
*R*_int_ = 0.019

#### Refinement

*R*[*F* ^2^ > 2σ(*F* ^2^)] = 0.034
*wR*(*F* ^2^) = 0.074
*S* = 1.04
9009 reflections
642 parameters
1 restraint
H-atom parameters constrained
Δρ_max_ = 1.40 e Å^−3^
Δρ_min_ = −0.46 e Å^−3^
Absolute structure: Flack (1983 ▶), 3943 Friedel pairs
Flack parameter: 0.029 (10)

### 

Data collection: *SMART* (Bruker, 2007 ▶); cell refinement: *SAINT* (Bruker, 2007 ▶); data reduction: *SAINT*; program(s) used to solve structure: *SHELXS97* (Sheldrick, 2008 ▶); program(s) used to refine structure: *SHELXL97* (Sheldrick, 2008 ▶); molecular graphics: *SHELXTL* (Sheldrick, 2008 ▶); software used to prepare material for publication: *SHELXTL*.

## Supplementary Material

Crystal structure: contains datablocks global, I. DOI: 10.1107/S1600536809030700/hy2214sup1.cif

Structure factors: contains datablocks I. DOI: 10.1107/S1600536809030700/hy2214Isup2.hkl

Additional supplementary materials:  crystallographic information; 3D view; checkCIF report

## Figures and Tables

**Table 1:** Selected bond lengths (Å)

Gd1—O1	2.338 (4)
Gd1—O2	2.371 (5)
Gd1—O3	2.356 (2)
Gd1—O4	2.340 (3)
Gd1—O5	2.361 (5)
Gd1—O6	2.351 (4)
Gd1—N1	2.601 (3)
Gd1—N2	2.587 (4)

**Fig. 1.**
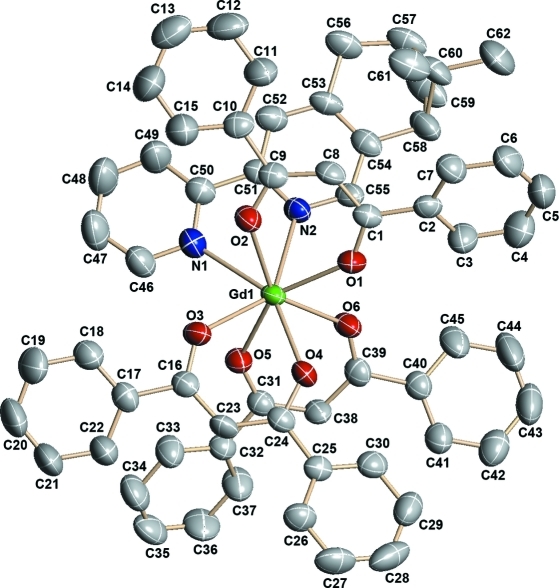
The molecular structure of the title compound. Displacement ellipsoids are drawn at the 30% probability level. H atoms have been omitted for clarity.

**Fig. 2.**
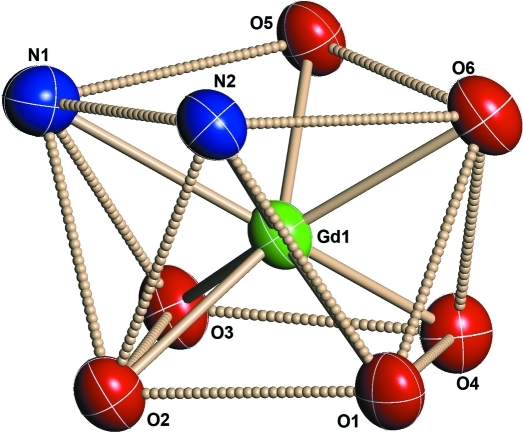
Coordination geometry of GdIII atom in the title compound.

## supplementary crystallographic information

### Comment

In the coordination chemistry field, chiral ligands play a key role in designing noncentrosymmetric complexes. In particular, many useful physical properties such as ferroelectricity, piezoelectricity, magneto-chiral dichroism and second harmony generation *etc*. are only based on noncentrosymmetric crystal lattices (Kaneko *et al.*, 2006; Kimura *et al.*, 2003). An effective and facile method to prepare such kind of complexes is the introducing of chiral motifs in molecule. Based on this strategy, as a continuance of our research (Li *et al.*, 2007), we employed a chiral ligands *L_S,S_*, (+)7,7-dimethyl-3-(2-pyridyl)-5,6,7,8-tetrahydro-6,8-methanoisoquinoline, to react with Gd(dbm)_3_.2H_2_O (dbm = dibenzoylmethanide), and an enantiomerically pure compound with formula Gd(dbm)_3_*L*_S,_*_S_* was obtained.

As shown in Fig. 1, the title compound contains three β-diketonate ligands, a chiral bipyridine derivative ligand and a Gd^III^ atom, which is isostructural with our previously reported Eu(dbm)_3_*L_S,S_* (Li *et al.*, 2007). The Gd^III^ atom is coordinated by six O atoms from three β-diketonate ligands and two N atoms from a chiral bipyridine derivative in an eight-coordinated distorted square-antiprismatic environment. The Gd—O bond distances rang from 2.338 (4) to 2.371 (5) Å, while two Gd—N bond distances are 2.587 (4) and 2.601 (3) Å. The two square planes defined by O5, O6, N1, N2 (top face) and O1, O2, O3, O4 (bottom face) (Fig.2) with mean deviations of 0.121 (3) and 0.063 (3) Å, respectively, make a dihedral angle of 1.40 (1)°. The title compound crystallizes in the chiral space group *P*2_1_ and the chirality is dominated by two chiral centers (C57 and C58) of bipyridine derivative.

### Experimental

The chiral ligand *L_S,S_* was prepared by the documented procedures (Hayoz & Zelewsky, 1992) and Gd(dbm)_3_.2H_2_O was synthesized according to literature method (Carles & Ohlmann, 1965). A solution of Gd(dbm)_3_.2H_2_O (0.086 g, 0.1 mmol) in ethanol was added to a solution of *L_S,S_* in acetone. The mixture was kept at room temperature. Yellow single crystals of the title compound, suitable for X-ray analysis, were obtained in 79% yield by slow evaporation of the solvent over two weeks. Analysis calculated for C_62_H_51_GdN_2_O_6_: C 69.00, H 4.77, N 2.60%; found: C 71.02, H 4.67, N 2.86%.

### Refinement

H atoms were positioned geometrically and treated as riding atoms, with C—H = 0.93 (aromatic), 0.98 (tertiary), 0.97 (methylene) and 0.96 (methyl) Å and with *U*_iso_(H) = 1.2(1.5 for methyl)*U*_eq_(C). The highest residual electron density was found 0.92 Å from Gd1 and the deepest hole 0.52 Å from H55.

### Crystal data

**Table:**

[Gd(C_15_H_11_O_2_)_3_(C_17_H_18_N_2_)]	*F*(000) = 1098
*M**_r_* = 1077.30	*D*_x_ = 1.418 Mg m^−^^3^
Monoclinic, *P*2_1_	Mo *K*α radiation, λ = 0.71073 Å
Hall symbol: P 2yb	Cell parameters from 13247 reflections
*a* = 9.5303 (19) Å	θ = 2.5–22.5°
*b* = 20.814 (4) Å	µ = 1.37 mm^−^^1^
*c* = 12.735 (2) Å	*T* = 298 K
β = 92.421 (4)°	Block, yellow
*V* = 2523.9 (8) Å^3^	0.30 × 0.24 × 0.22 mm
*Z* = 2	

### Data collection

**Table:**

Bruker SMART APEX CCD diffractometer	9009 independent reflections
Radiation source: fine-focus sealed tube	8750 reflections with *I* > 2σ(*I*)
graphite	*R*_int_ = 0.019
φ and ω scans	θ_max_ = 26.0°, θ_min_ = 1.9°
Absorption correction: multi-scan (*SADABS*; Bruker, 2001)	*h* = −11→8
*T*_min_ = 0.680, *T*_max_ = 0.742	*k* = −25→24
13247 measured reflections	*l* = −15→15

### Refinement

**Table:**

Refinement on *F*^2^	Secondary atom site location: difference Fourier map
Least-squares matrix: full	Hydrogen site location: inferred from neighbouring sites
*R*[*F*^2^ > 2σ(*F*^2^)] = 0.034	H-atom parameters constrained
*wR*(*F*^2^) = 0.074	*w* = 1/[σ^2^(*F*_o_^2^) + (0.015*P*)^2^ + 1.990*P*] where *P* = (*F*_o_^2^ + 2*F*_c_^2^)/3
*S* = 1.04	(Δ/σ)_max_ = 0.002
9009 reflections	Δρ_max_ = 1.40 e Å^−^^3^
642 parameters	Δρ_min_ = −0.46 e Å^−^^3^
1 restraint	Absolute structure: Flack (1983), 3943 Friedel pairs
Primary atom site location: structure-invariant direct methods	Flack parameter: 0.029 (10)

### Fractional atomic coordinates and isotropic or equivalent isotropic displacement parameters (Å^2^)

**Table:**

	*x*	*y*	*z*	*U*_iso_*/*U*_eq_	
C1	0.1984 (6)	0.2577 (3)	0.6357 (6)	0.0406 (15)	
C2	0.2001 (6)	0.2429 (3)	0.5220 (6)	0.0450 (16)	
C3	0.2043 (8)	0.2949 (3)	0.4518 (6)	0.0657 (18)	
H3	0.2027	0.3369	0.4766	0.079*	
C4	0.2108 (10)	0.2829 (5)	0.3455 (8)	0.086 (3)	
H4	0.2187	0.3170	0.2989	0.103*	
C5	0.2058 (9)	0.2200 (8)	0.3072 (6)	0.088 (4)	
H5	0.2060	0.2125	0.2352	0.106*	
C6	0.2005 (7)	0.1701 (4)	0.3742 (5)	0.0768 (18)	
H6	0.1984	0.1282	0.3486	0.092*	
C7	0.1983 (6)	0.1813 (3)	0.4818 (4)	0.0588 (13)	
H7	0.1955	0.1466	0.5276	0.071*	
C8	0.2526 (5)	0.2128 (2)	0.7094 (4)	0.0481 (11)	
H8	0.2797	0.1728	0.6845	0.058*	
C9	0.2687 (5)	0.2240 (2)	0.8176 (4)	0.0450 (11)	
C10	0.3456 (5)	0.1766 (2)	0.8871 (4)	0.0499 (11)	
C11	0.4313 (7)	0.1291 (3)	0.8488 (5)	0.0734 (17)	
H11	0.4386	0.1245	0.7766	0.088*	
C12	0.5056 (8)	0.0886 (4)	0.9156 (7)	0.091 (2)	
H12	0.5634	0.0573	0.8882	0.109*	
C13	0.4954 (7)	0.0937 (4)	1.0210 (6)	0.087 (2)	
H13	0.5466	0.0665	1.0660	0.104*	
C14	0.4097 (10)	0.1390 (5)	1.0604 (6)	0.105 (3)	
H14	0.4009	0.1421	1.1327	0.126*	
C15	0.3353 (8)	0.1808 (4)	0.9942 (5)	0.085 (2)	
H15	0.2779	0.2119	1.0224	0.102*	
C16	−0.0584 (5)	0.4176 (2)	0.9698 (4)	0.0452 (10)	
C17	−0.0895 (5)	0.4255 (3)	1.0831 (4)	0.0517 (12)	
C18	−0.0309 (5)	0.3839 (5)	1.1566 (3)	0.0616 (11)	
H18	0.0312	0.3525	1.1353	0.074*	
C19	−0.0621 (7)	0.3877 (6)	1.2611 (4)	0.0850 (19)	
H19	−0.0240	0.3580	1.3089	0.102*	
C20	−0.1479 (9)	0.4345 (4)	1.2944 (5)	0.098 (3)	
H20	−0.1701	0.4366	1.3648	0.118*	
C21	−0.2013 (9)	0.4781 (4)	1.2251 (5)	0.100 (3)	
H21	−0.2575	0.5111	1.2489	0.120*	
C22	−0.1742 (7)	0.4746 (3)	1.1202 (4)	0.0735 (18)	
H22	−0.2123	0.5050	1.0737	0.088*	
C23	−0.1440 (5)	0.4482 (3)	0.8941 (4)	0.0500 (12)	
H23	−0.2143	0.4750	0.9172	0.060*	
C24	−0.1316 (5)	0.4415 (2)	0.7853 (4)	0.0436 (10)	
C25	−0.2364 (6)	0.4742 (2)	0.7126 (4)	0.0428 (11)	
C26	−0.3633 (6)	0.4979 (3)	0.7450 (5)	0.0586 (14)	
H26	−0.3861	0.4936	0.8150	0.070*	
C27	−0.4553 (6)	0.5274 (3)	0.6756 (6)	0.0751 (18)	
H27	−0.5402	0.5432	0.6983	0.090*	
C28	−0.4226 (8)	0.5337 (3)	0.5724 (6)	0.085 (2)	
H28	−0.4856	0.5537	0.5251	0.102*	
C29	−0.2989 (9)	0.5112 (4)	0.5386 (5)	0.073 (2)	
H29	−0.2774	0.5155	0.4684	0.087*	
C30	−0.2042 (6)	0.4814 (3)	0.6091 (4)	0.0549 (12)	
H30	−0.1189	0.4664	0.5861	0.066*	
C31	0.1593 (5)	0.5411 (2)	0.8180 (4)	0.0431 (10)	
C32	0.1256 (5)	0.5972 (2)	0.8867 (4)	0.0442 (10)	
C33	0.0830 (7)	0.5865 (3)	0.9877 (4)	0.0640 (15)	
H33	0.0794	0.5447	1.0135	0.077*	
C34	0.0461 (7)	0.6369 (3)	1.0503 (5)	0.0794 (19)	
H34	0.0151	0.6287	1.1172	0.095*	
C35	0.0539 (7)	0.6987 (3)	1.0163 (5)	0.0721 (17)	
H35	0.0287	0.7325	1.0594	0.086*	
C36	0.0990 (8)	0.7100 (3)	0.9186 (5)	0.0818 (19)	
H36	0.1065	0.7521	0.8949	0.098*	
C37	0.1340 (8)	0.6599 (3)	0.8537 (5)	0.0719 (16)	
H37	0.1636	0.6687	0.7865	0.086*	
C38	0.1460 (5)	0.5486 (2)	0.7089 (4)	0.0459 (11)	
H38	0.1079	0.5868	0.6827	0.055*	
C39	0.1864 (7)	0.5022 (3)	0.6366 (6)	0.0470 (16)	
C40	0.1748 (7)	0.5178 (3)	0.5201 (5)	0.0479 (16)	
C41	0.0875 (6)	0.5645 (3)	0.4778 (4)	0.0594 (13)	
H41	0.0310	0.5879	0.5217	0.071*	
C42	0.0823 (8)	0.5772 (3)	0.3717 (5)	0.0764 (18)	
H42	0.0209	0.6082	0.3439	0.092*	
C43	0.1682 (12)	0.5441 (7)	0.3072 (6)	0.094 (4)	
H43	0.1645	0.5523	0.2354	0.112*	
C44	0.2580 (12)	0.4996 (5)	0.3476 (7)	0.104 (4)	
H44	0.3186	0.4783	0.3039	0.125*	
C45	0.2603 (9)	0.4853 (3)	0.4538 (5)	0.073 (2)	
H45	0.3202	0.4535	0.4806	0.088*	
C46	0.3468 (6)	0.3970 (3)	1.0578 (4)	0.066 (2)	
H46	0.2635	0.4192	1.0667	0.080*	
C47	0.4284 (6)	0.3824 (7)	1.1449 (4)	0.0866 (17)	
H47	0.4014	0.3945	1.2114	0.104*	
C48	0.5500 (8)	0.3499 (4)	1.1324 (5)	0.099 (3)	
H48	0.6077	0.3394	1.1906	0.119*	
C49	0.5869 (7)	0.3329 (4)	1.0343 (5)	0.084 (2)	
H49	0.6696	0.3103	1.0250	0.100*	
C50	0.5004 (5)	0.3495 (3)	0.9479 (4)	0.0518 (12)	
C51	0.5374 (5)	0.3343 (2)	0.8389 (4)	0.0452 (11)	
C52	0.6568 (5)	0.3007 (3)	0.8165 (4)	0.0565 (13)	
H52	0.7152	0.2850	0.8710	0.068*	
C53	0.6904 (6)	0.2902 (3)	0.7133 (5)	0.0504 (13)	
C54	0.6027 (5)	0.3156 (2)	0.6349 (4)	0.0507 (12)	
C55	0.4842 (5)	0.3477 (2)	0.6641 (4)	0.0479 (11)	
H55	0.4246	0.3639	0.6109	0.057*	
C56	0.8148 (5)	0.2501 (3)	0.6833 (5)	0.0644 (15)	
H56A	0.8062	0.2070	0.7115	0.077*	
H56B	0.9009	0.2689	0.7127	0.077*	
C57	0.8199 (6)	0.2472 (3)	0.5639 (5)	0.0682 (16)	
H57	0.8995	0.2231	0.5375	0.082*	
C58	0.6414 (7)	0.3041 (3)	0.5241 (5)	0.0710 (17)	
H58	0.5822	0.3245	0.4688	0.085*	
C59	0.8038 (8)	0.3159 (3)	0.5210 (6)	0.086 (2)	
H59A	0.8379	0.3218	0.4510	0.104*	
H59B	0.8392	0.3490	0.5687	0.104*	
C60	0.6760 (7)	0.2310 (3)	0.5093 (5)	0.0646 (17)	
C61	0.5839 (6)	0.1819 (3)	0.5623 (6)	0.0771 (18)	
H61A	0.4949	0.1790	0.5243	0.116*	
H61B	0.5694	0.1951	0.6333	0.116*	
H61C	0.6292	0.1407	0.5626	0.116*	
C62	0.6851 (10)	0.2136 (4)	0.3939 (6)	0.106 (3)	
H62A	0.7325	0.2472	0.3580	0.160*	
H62B	0.5921	0.2083	0.3631	0.160*	
H62C	0.7363	0.1741	0.3878	0.160*	
Gd1	0.191494 (18)	0.38195 (2)	0.805878 (13)	0.03808 (6)	
N1	0.3797 (3)	0.3811 (4)	0.9598 (2)	0.0506 (7)	
N2	0.4487 (4)	0.35710 (18)	0.7629 (3)	0.0438 (9)	
O1	0.1581 (4)	0.31363 (18)	0.6612 (3)	0.0491 (9)	
O2	0.2229 (5)	0.2740 (2)	0.8617 (4)	0.0487 (11)	
O3	0.0450 (3)	0.3816 (3)	0.95033 (19)	0.0506 (6)	
O4	−0.0349 (3)	0.41022 (15)	0.7441 (2)	0.0481 (8)	
O5	0.1972 (5)	0.4897 (2)	0.8640 (4)	0.0477 (11)	
O6	0.2329 (4)	0.44748 (18)	0.6600 (3)	0.0498 (9)	

### Atomic displacement parameters (Å^2^)

**Table:**

	*U*^11^	*U*^22^	*U*^33^	*U*^12^	*U*^13^	*U*^23^
C1	0.034 (3)	0.034 (3)	0.053 (4)	−0.002 (2)	0.004 (3)	−0.005 (3)
C2	0.035 (3)	0.048 (4)	0.052 (4)	0.008 (2)	−0.001 (2)	−0.001 (3)
C3	0.087 (5)	0.054 (4)	0.056 (4)	0.012 (3)	0.002 (3)	0.002 (3)
C4	0.105 (6)	0.092 (7)	0.061 (4)	0.011 (5)	0.008 (4)	0.017 (4)
C5	0.106 (7)	0.110 (10)	0.047 (5)	0.035 (6)	0.007 (4)	−0.003 (4)
C6	0.086 (5)	0.083 (5)	0.061 (4)	0.021 (4)	−0.002 (3)	−0.023 (3)
C7	0.060 (3)	0.057 (3)	0.060 (3)	0.004 (3)	0.004 (3)	−0.008 (3)
C8	0.053 (3)	0.034 (3)	0.058 (3)	0.007 (2)	0.010 (2)	−0.005 (2)
C9	0.044 (3)	0.037 (3)	0.055 (3)	−0.0030 (19)	0.010 (2)	0.005 (2)
C10	0.051 (3)	0.040 (3)	0.059 (3)	−0.001 (2)	0.006 (2)	0.008 (2)
C11	0.088 (4)	0.064 (4)	0.069 (4)	0.024 (3)	0.011 (3)	0.013 (3)
C12	0.090 (5)	0.075 (5)	0.109 (6)	0.035 (4)	0.015 (4)	0.027 (4)
C13	0.073 (4)	0.090 (5)	0.097 (5)	0.011 (4)	0.000 (4)	0.037 (4)
C14	0.130 (7)	0.124 (8)	0.063 (4)	0.045 (6)	0.002 (4)	0.024 (4)
C15	0.098 (5)	0.090 (5)	0.067 (4)	0.031 (4)	0.009 (3)	0.006 (3)
C16	0.051 (3)	0.037 (3)	0.049 (3)	0.001 (2)	0.011 (2)	0.005 (2)
C17	0.053 (3)	0.056 (3)	0.047 (3)	−0.002 (2)	0.014 (2)	−0.002 (2)
C18	0.069 (3)	0.060 (3)	0.058 (2)	0.009 (5)	0.014 (2)	0.004 (5)
C19	0.111 (4)	0.092 (5)	0.054 (3)	0.017 (6)	0.024 (3)	0.021 (5)
C20	0.142 (7)	0.108 (6)	0.047 (3)	0.030 (5)	0.032 (4)	−0.004 (4)
C21	0.138 (7)	0.110 (6)	0.054 (3)	0.056 (5)	0.023 (4)	−0.010 (4)
C22	0.093 (5)	0.077 (4)	0.051 (3)	0.029 (3)	0.015 (3)	−0.001 (3)
C23	0.040 (2)	0.059 (3)	0.051 (3)	0.015 (2)	0.010 (2)	−0.007 (2)
C24	0.044 (3)	0.038 (3)	0.049 (3)	−0.0015 (19)	0.008 (2)	−0.005 (2)
C25	0.037 (3)	0.040 (3)	0.051 (3)	−0.002 (2)	0.000 (2)	−0.006 (2)
C26	0.045 (3)	0.063 (4)	0.068 (4)	0.004 (2)	0.001 (3)	−0.004 (3)
C27	0.047 (3)	0.072 (4)	0.105 (5)	0.015 (3)	−0.010 (3)	−0.009 (4)
C28	0.088 (5)	0.060 (4)	0.103 (6)	0.016 (4)	−0.043 (4)	0.002 (4)
C29	0.103 (6)	0.061 (5)	0.053 (4)	0.007 (4)	−0.012 (3)	0.004 (3)
C30	0.059 (3)	0.045 (3)	0.060 (3)	0.006 (2)	0.001 (3)	−0.005 (3)
C31	0.043 (3)	0.039 (3)	0.048 (3)	−0.0010 (19)	0.009 (2)	−0.001 (2)
C32	0.045 (3)	0.041 (3)	0.047 (2)	0.0007 (19)	0.002 (2)	−0.003 (2)
C33	0.089 (4)	0.052 (3)	0.052 (3)	0.002 (3)	0.009 (3)	0.002 (2)
C34	0.104 (5)	0.079 (5)	0.056 (3)	0.007 (4)	0.015 (3)	−0.019 (3)
C35	0.075 (4)	0.067 (4)	0.075 (4)	0.012 (3)	0.002 (3)	−0.030 (3)
C36	0.122 (6)	0.038 (3)	0.086 (4)	0.012 (3)	0.008 (4)	−0.004 (3)
C37	0.111 (5)	0.046 (3)	0.060 (3)	0.003 (3)	0.011 (3)	0.001 (3)
C38	0.061 (3)	0.031 (2)	0.046 (3)	0.007 (2)	0.010 (2)	0.0083 (19)
C39	0.047 (4)	0.051 (4)	0.044 (4)	−0.003 (3)	0.014 (3)	0.004 (3)
C40	0.054 (4)	0.045 (4)	0.045 (3)	−0.002 (3)	0.011 (3)	0.003 (3)
C41	0.073 (4)	0.058 (4)	0.048 (3)	0.005 (3)	0.010 (3)	0.005 (2)
C42	0.100 (5)	0.070 (4)	0.058 (4)	−0.001 (4)	−0.003 (3)	0.013 (3)
C43	0.152 (9)	0.085 (8)	0.044 (5)	0.008 (6)	0.006 (5)	0.004 (4)
C44	0.174 (10)	0.088 (7)	0.054 (5)	0.033 (7)	0.037 (6)	−0.005 (4)
C45	0.116 (6)	0.048 (4)	0.057 (4)	0.016 (4)	0.019 (4)	−0.002 (3)
C46	0.063 (3)	0.078 (6)	0.058 (3)	0.009 (3)	0.007 (2)	−0.015 (3)
C47	0.087 (4)	0.120 (5)	0.052 (3)	0.001 (7)	0.003 (3)	−0.018 (7)
C48	0.090 (5)	0.151 (8)	0.056 (3)	0.026 (5)	−0.009 (3)	−0.006 (4)
C49	0.064 (4)	0.125 (6)	0.061 (4)	0.030 (4)	−0.004 (3)	−0.007 (4)
C50	0.046 (3)	0.052 (3)	0.058 (3)	0.002 (2)	0.007 (2)	−0.003 (2)
C51	0.040 (2)	0.040 (3)	0.055 (3)	0.0012 (19)	0.006 (2)	−0.003 (2)
C52	0.047 (3)	0.056 (3)	0.066 (3)	0.009 (2)	0.000 (2)	−0.008 (3)
C53	0.034 (3)	0.046 (3)	0.072 (4)	0.005 (2)	0.012 (3)	−0.009 (3)
C54	0.053 (3)	0.039 (3)	0.061 (3)	−0.003 (2)	0.022 (2)	−0.003 (2)
C55	0.053 (3)	0.040 (3)	0.051 (3)	0.011 (2)	0.012 (2)	0.008 (2)
C56	0.038 (3)	0.070 (4)	0.086 (4)	0.005 (2)	0.011 (3)	−0.018 (3)
C57	0.049 (3)	0.059 (4)	0.100 (5)	0.002 (3)	0.033 (3)	−0.010 (3)
C58	0.090 (4)	0.066 (4)	0.060 (3)	0.020 (3)	0.035 (3)	0.002 (3)
C59	0.099 (5)	0.061 (4)	0.105 (5)	−0.003 (3)	0.063 (4)	−0.006 (4)
C60	0.070 (4)	0.059 (4)	0.066 (4)	0.012 (3)	0.024 (3)	−0.018 (3)
C61	0.055 (3)	0.067 (4)	0.110 (5)	−0.006 (3)	0.009 (3)	−0.023 (4)
C62	0.133 (7)	0.107 (6)	0.081 (5)	0.042 (5)	0.020 (4)	−0.030 (4)
Gd1	0.04094 (10)	0.03390 (10)	0.04003 (9)	0.00921 (12)	0.00911 (6)	0.00091 (15)
N1	0.0537 (19)	0.0507 (19)	0.0479 (17)	−0.004 (3)	0.0085 (14)	−0.006 (4)
N2	0.044 (2)	0.043 (2)	0.046 (2)	0.0035 (15)	0.0115 (17)	0.0020 (15)
O1	0.054 (2)	0.041 (2)	0.052 (2)	0.0123 (17)	0.0020 (18)	−0.0002 (15)
O2	0.060 (2)	0.042 (2)	0.045 (3)	0.0052 (18)	0.0094 (19)	−0.001 (2)
O3	0.0519 (15)	0.0512 (16)	0.0496 (14)	0.016 (3)	0.0123 (12)	0.007 (3)
O4	0.0523 (18)	0.0517 (19)	0.0410 (16)	0.0099 (14)	0.0107 (14)	−0.0060 (13)
O5	0.060 (3)	0.037 (2)	0.046 (3)	0.0077 (17)	0.0071 (18)	0.0028 (19)
O6	0.061 (2)	0.042 (2)	0.0484 (19)	0.0111 (18)	0.0178 (18)	0.0027 (15)

### Geometric parameters (Å, °)

**Table:**

C1—O1	1.273 (7)	C35—H35	0.9300
C1—C8	1.408 (8)	C36—C37	1.381 (8)
C1—C2	1.480 (10)	C36—H36	0.9300
C2—C7	1.381 (9)	C37—H37	0.9300
C2—C3	1.406 (10)	C38—C39	1.399 (9)
C3—C4	1.381 (12)	C38—H38	0.9300
C3—H3	0.9300	C39—O6	1.253 (8)
C4—C5	1.397 (18)	C39—C40	1.519 (10)
C4—H4	0.9300	C40—C45	1.376 (9)
C5—C6	1.346 (15)	C40—C41	1.374 (9)
C5—H5	0.9300	C41—C42	1.375 (8)
C6—C7	1.391 (8)	C41—H41	0.9300
C6—H6	0.9300	C42—C43	1.370 (13)
C7—H7	0.9300	C42—H42	0.9300
C8—C9	1.400 (7)	C43—C44	1.348 (16)
C8—H8	0.9300	C43—H43	0.9300
C9—O2	1.269 (6)	C44—C45	1.384 (11)
C9—C10	1.498 (7)	C44—H44	0.9300
C10—C15	1.374 (8)	C45—H45	0.9300
C10—C11	1.384 (8)	C46—N1	1.340 (6)
C11—C12	1.372 (9)	C46—C47	1.362 (8)
C11—H11	0.9300	C46—H46	0.9300
C12—C13	1.355 (10)	C47—C48	1.356 (11)
C12—H12	0.9300	C47—H47	0.9300
C13—C14	1.357 (11)	C48—C49	1.360 (9)
C13—H13	0.9300	C48—H48	0.9300
C14—C15	1.386 (9)	C49—C50	1.390 (8)
C14—H14	0.9300	C49—H49	0.9300
C15—H15	0.9300	C50—N1	1.340 (7)
C16—O3	1.269 (6)	C50—C51	1.481 (7)
C16—C23	1.392 (7)	C51—N2	1.344 (6)
C16—C17	1.495 (6)	C51—C52	1.376 (7)
C17—C18	1.376 (8)	C52—C53	1.383 (8)
C17—C22	1.397 (7)	C52—H52	0.9300
C18—C19	1.378 (6)	C53—C54	1.379 (8)
C18—H18	0.9300	C53—C56	1.513 (7)
C19—C20	1.352 (11)	C54—C55	1.376 (7)
C19—H19	0.9300	C54—C58	1.493 (7)
C20—C21	1.350 (10)	C55—N2	1.331 (6)
C20—H20	0.9300	C55—H55	0.9300
C21—C22	1.373 (8)	C56—C57	1.525 (9)
C21—H21	0.9300	C56—H56A	0.9700
C22—H22	0.9300	C56—H56B	0.9700
C23—C24	1.402 (6)	C57—C59	1.537 (9)
C23—H23	0.9300	C57—C60	1.548 (9)
C24—O4	1.261 (5)	C57—H57	0.9800
C24—C25	1.496 (7)	C58—C59	1.570 (10)
C25—C30	1.375 (8)	C58—C60	1.569 (9)
C25—C26	1.385 (7)	C58—H58	0.9800
C26—C27	1.365 (8)	C59—H59A	0.9700
C26—H26	0.9300	C59—H59B	0.9700
C27—C28	1.370 (10)	C60—C62	1.519 (9)
C27—H27	0.9300	C60—C61	1.524 (10)
C28—C29	1.356 (10)	C61—H61A	0.9600
C28—H28	0.9300	C61—H61B	0.9600
C29—C30	1.391 (9)	C61—H61C	0.9600
C29—H29	0.9300	C62—H62A	0.9600
C30—H30	0.9300	C62—H62B	0.9600
C31—O5	1.266 (6)	C62—H62C	0.9600
C31—C38	1.399 (6)	Gd1—O1	2.338 (4)
C31—C32	1.502 (6)	Gd1—O2	2.371 (5)
C32—C37	1.374 (8)	Gd1—O3	2.356 (2)
C32—C33	1.383 (7)	Gd1—O4	2.340 (3)
C33—C34	1.374 (8)	Gd1—O5	2.361 (5)
C33—H33	0.9300	Gd1—O6	2.351 (4)
C34—C35	1.358 (9)	Gd1—N1	2.601 (3)
C34—H34	0.9300	Gd1—N2	2.587 (4)
C35—C36	1.355 (9)		
			
O1—C1—C8	122.9 (6)	C43—C42—H42	120.2
O1—C1—C2	117.1 (6)	C41—C42—H42	120.2
C8—C1—C2	119.7 (6)	C44—C43—C42	120.2 (7)
C7—C2—C3	118.6 (6)	C44—C43—H43	119.9
C7—C2—C1	123.7 (6)	C42—C43—H43	119.9
C3—C2—C1	117.7 (6)	C43—C44—C45	120.2 (8)
C4—C3—C2	119.2 (7)	C43—C44—H44	119.9
C4—C3—H3	120.4	C45—C44—H44	119.9
C2—C3—H3	120.4	C40—C45—C44	120.6 (8)
C3—C4—C5	120.6 (8)	C40—C45—H45	119.7
C3—C4—H4	119.7	C44—C45—H45	119.7
C5—C4—H4	119.7	N1—C46—C47	123.7 (6)
C6—C5—C4	120.3 (7)	N1—C46—H46	118.1
C6—C5—H5	119.9	C47—C46—H46	118.1
C4—C5—H5	119.9	C48—C47—C46	118.5 (6)
C5—C6—C7	119.8 (8)	C48—C47—H47	120.8
C5—C6—H6	120.1	C46—C47—H47	120.8
C7—C6—H6	120.1	C47—C48—C49	119.5 (6)
C2—C7—C6	121.4 (6)	C47—C48—H48	120.2
C2—C7—H7	119.3	C49—C48—H48	120.2
C6—C7—H7	119.3	C48—C49—C50	119.7 (6)
C9—C8—C1	124.5 (5)	C48—C49—H49	120.2
C9—C8—H8	117.7	C50—C49—H49	120.2
C1—C8—H8	117.7	N1—C50—C49	121.0 (5)
O2—C9—C8	123.2 (5)	N1—C50—C51	116.8 (4)
O2—C9—C10	116.7 (5)	C49—C50—C51	122.2 (5)
C8—C9—C10	120.1 (4)	N2—C51—C52	122.1 (4)
C15—C10—C11	117.6 (5)	N2—C51—C50	115.6 (4)
C15—C10—C9	119.4 (5)	C52—C51—C50	122.3 (5)
C11—C10—C9	123.0 (5)	C51—C52—C53	120.2 (5)
C12—C11—C10	121.1 (6)	C51—C52—H52	119.9
C12—C11—H11	119.4	C53—C52—H52	119.9
C10—C11—H11	119.4	C54—C53—C52	118.1 (5)
C13—C12—C11	120.6 (7)	C54—C53—C56	119.1 (5)
C13—C12—H12	119.7	C52—C53—C56	122.8 (5)
C11—C12—H12	119.7	C55—C54—C53	118.0 (5)
C12—C13—C14	119.4 (6)	C55—C54—C58	124.8 (5)
C12—C13—H13	120.3	C53—C54—C58	117.2 (5)
C14—C13—H13	120.3	N2—C55—C54	124.8 (5)
C13—C14—C15	120.8 (7)	N2—C55—H55	117.6
C13—C14—H14	119.6	C54—C55—H55	117.6
C15—C14—H14	119.6	C53—C56—C57	109.4 (5)
C10—C15—C14	120.5 (7)	C53—C56—H56A	109.8
C10—C15—H15	119.8	C57—C56—H56A	109.8
C14—C15—H15	119.8	C53—C56—H56B	109.8
O3—C16—C23	124.9 (4)	C57—C56—H56B	109.8
O3—C16—C17	116.2 (4)	H56A—C56—H56B	108.2
C23—C16—C17	118.9 (4)	C56—C57—C59	108.1 (5)
C18—C17—C22	117.0 (5)	C56—C57—C60	113.0 (4)
C18—C17—C16	119.7 (5)	C59—C57—C60	88.2 (5)
C22—C17—C16	123.2 (5)	C56—C57—H57	114.8
C19—C18—C17	121.5 (8)	C59—C57—H57	114.8
C19—C18—H18	119.2	C60—C57—H57	114.8
C17—C18—H18	119.2	C54—C58—C59	106.4 (6)
C20—C19—C18	120.2 (8)	C54—C58—C60	109.3 (5)
C20—C19—H19	119.9	C59—C58—C60	86.3 (5)
C18—C19—H19	119.9	C54—C58—H58	116.9
C19—C20—C21	119.7 (6)	C59—C58—H58	116.9
C19—C20—H20	120.2	C60—C58—H58	116.9
C21—C20—H20	120.2	C57—C59—C58	85.9 (5)
C20—C21—C22	121.3 (6)	C57—C59—H59A	114.3
C20—C21—H21	119.4	C58—C59—H59A	114.3
C22—C21—H21	119.4	C57—C59—H59B	114.3
C21—C22—C17	120.2 (6)	C58—C59—H59B	114.3
C21—C22—H22	119.9	H59A—C59—H59B	111.5
C17—C22—H22	119.9	C62—C60—C61	109.0 (6)
C16—C23—C24	124.6 (4)	C62—C60—C57	113.6 (6)
C16—C23—H23	117.7	C61—C60—C57	117.6 (6)
C24—C23—H23	117.7	C62—C60—C58	111.6 (6)
O4—C24—C23	123.7 (4)	C61—C60—C58	118.0 (5)
O4—C24—C25	117.3 (4)	C57—C60—C58	85.6 (5)
C23—C24—C25	119.0 (4)	C60—C61—H61A	109.5
C30—C25—C26	118.7 (5)	C60—C61—H61B	109.5
C30—C25—C24	118.2 (5)	H61A—C61—H61B	109.5
C26—C25—C24	123.1 (5)	C60—C61—H61C	109.5
C27—C26—C25	120.8 (6)	H61A—C61—H61C	109.5
C27—C26—H26	119.6	H61B—C61—H61C	109.5
C25—C26—H26	119.6	C60—C62—H62A	109.5
C26—C27—C28	119.9 (6)	C60—C62—H62B	109.5
C26—C27—H27	120.0	H62A—C62—H62B	109.5
C28—C27—H27	120.0	C60—C62—H62C	109.5
C29—C28—C27	120.5 (6)	H62A—C62—H62C	109.5
C29—C28—H28	119.8	H62B—C62—H62C	109.5
C27—C28—H28	119.8	O1—Gd1—O4	78.18 (11)
C28—C29—C30	119.9 (7)	O1—Gd1—O6	75.64 (11)
C28—C29—H29	120.0	O4—Gd1—O6	76.79 (12)
C30—C29—H29	120.0	O1—Gd1—O3	123.22 (16)
C25—C30—C29	120.2 (6)	O4—Gd1—O3	72.24 (10)
C25—C30—H30	119.9	O6—Gd1—O3	138.02 (15)
C29—C30—H30	119.9	O1—Gd1—O5	145.58 (14)
O5—C31—C38	124.4 (4)	O4—Gd1—O5	82.84 (14)
O5—C31—C32	116.9 (4)	O6—Gd1—O5	72.19 (14)
C38—C31—C32	118.7 (4)	O3—Gd1—O5	76.41 (19)
C37—C32—C33	117.5 (5)	O1—Gd1—O2	70.82 (14)
C37—C32—C31	122.9 (4)	O4—Gd1—O2	116.22 (14)
C33—C32—C31	119.7 (4)	O6—Gd1—O2	139.74 (14)
C34—C33—C32	120.6 (6)	O3—Gd1—O2	80.47 (18)
C34—C33—H33	119.7	O5—Gd1—O2	143.59 (9)
C32—C33—H33	119.7	O1—Gd1—N2	79.06 (12)
C35—C34—C33	121.3 (6)	O4—Gd1—N2	147.95 (11)
C35—C34—H34	119.3	O6—Gd1—N2	75.92 (12)
C33—C34—H34	119.3	O3—Gd1—N2	139.73 (11)
C36—C35—C34	118.7 (5)	O5—Gd1—N2	104.29 (14)
C36—C35—H35	120.6	O2—Gd1—N2	76.43 (13)
C34—C35—H35	120.6	O1—Gd1—N1	131.03 (17)
C35—C36—C37	120.8 (6)	O4—Gd1—N1	148.41 (12)
C35—C36—H36	119.6	O6—Gd1—N1	117.77 (16)
C37—C36—H36	119.6	O3—Gd1—N1	79.88 (9)
C32—C37—C36	121.0 (6)	O5—Gd1—N1	76.3 (2)
C32—C37—H37	119.5	O2—Gd1—N1	72.2 (2)
C36—C37—H37	119.5	N2—Gd1—N1	61.87 (11)
C31—C38—C39	124.1 (5)	C46—N1—C50	117.5 (4)
C31—C38—H38	117.9	C46—N1—Gd1	121.2 (3)
C39—C38—H38	117.9	C50—N1—Gd1	119.1 (3)
O6—C39—C38	125.1 (6)	C55—N2—C51	116.8 (4)
O6—C39—C40	115.9 (6)	C55—N2—Gd1	120.7 (3)
C38—C39—C40	118.9 (6)	C51—N2—Gd1	119.4 (3)
C45—C40—C41	118.2 (6)	C1—O1—Gd1	136.5 (4)
C45—C40—C39	118.3 (6)	C9—O2—Gd1	133.3 (4)
C41—C40—C39	123.5 (6)	C16—O3—Gd1	129.9 (3)
C42—C41—C40	121.1 (6)	C24—O4—Gd1	132.1 (3)
C42—C41—H41	119.4	C31—O5—Gd1	131.0 (4)
C40—C41—H41	119.4	C39—O6—Gd1	130.2 (4)
C43—C42—C41	119.6 (7)		
